# Taming the Lion: How Perceived Worth Buffers the Detrimental Influence of Power on Aggression and Conflict

**DOI:** 10.3389/fpsyg.2018.00858

**Published:** 2018-06-14

**Authors:** Mario Weick, Milica Vasiljevic, Constantine Sedikides

**Affiliations:** ^1^School of Psychology, University of Kent, Canterbury, United Kingdom; ^2^Cambridge Institute of Public Health, University of Cambridge, Cambridge, United Kingdom; ^3^Centre for Research on Self and Identity, Psychology Department, University of Southampton, Southampton, United Kingdom

**Keywords:** power, worth, self-esteem, status, aggression, conflict

## Abstract

Contrary to conventional wisdom, there is little empirical evidence that elevated power, by default, fuels conflict and aggression. Instead, previous studies have shown that extraneous factors that decrease powerholders’ perceived worth, making powerholders feel inferior or disrespected, seem to be necessary to ‘unleash’ power’s dark side and trigger aggression and conflict. However, this past work has largely neglected that power boosts individuals’ perceptions of worth, and as such these variables are not independent. The present research sought to address this oversight, thereby providing a more nuanced account of how perceived worth stifles aggression and conflict tendencies in powerholders. Focusing on *self-esteem* (Study 1) and *status* (Study 2) as two interrelated facets of perceived worth, we report primary and secondary data indicating that perceived worth acts as buffer and counters aggression as well as more general conflict tendencies in powerholders. By providing evidence for a suppression effect, the present findings go beyond the moderations identified in prior work and demonstrate that perceptions of worth are critical to understanding the link between power on the one hand, and aggression and conflict on the other. We conclude by discussing the social regulatory function of perceived worth in hierarchical relations.

## Introduction

Since power over human beings is shown in making them do what they would rather not do, the man who is actuated by love of power is more apt to inflict pain than to permit pleasure.

– Betrand Russell, Nobel Lecture, 1950

The notion that power transforms people into fiends is pervasive and a unifying theme in Plato’s *Republic*, Shakespeare’s *Macbeth*, and Machiavelli’s *The Prince*. The image of malevolent and coercive power-figures also resonates with Kipnis’s early studies on the corrupting effects of power and Zimbardo and colleagues’ prison experiment (Haney et al., 1973; Kipnis, 1976). However, sociological studies show that conflicts are often less, rather than more, common among rich and wealthy individuals as well as communities (Williams, 1984; Sampson et al., 1997; Browne et al., 1999). Contrary to Lord Acton’s famous assertion that absolute power corrupts absolutely, high levels of incidental power can sometimes prevent abuse (Sachdev and Bourhis, 1985) and reduce, rather than increase, vengeance in persons who are accustomed to power (Strelan et al., 2014). The present article seeks to clarify the vagarious relation between power on the one hand, and aggression/conflict on the other. We argue that the key to understanding this relation lies in the role of powerholders’ perceived worth, that is, the extent to which powerholders feel liked and respected in their own eyes (*self-esteem*) and in the eyes of others (*status*).

### How Power Facilitates Conflict and Aggression *in Theory*

Power implies control over outcomes and resources, and affords the ability to administer or withhold punishment (Keltner et al., 2003). Power predisposes individuals to take decisive actions (Galinsky et al., 2003), liberated from constraints and concerns over the consequences of such actions (Anderson and Galinsky, 2006; Overbeck et al., 2006). Perhaps unsurprisingly, then, power blunts individuals’ sensitivity to the feelings of others (Van Kleef et al., 2008; Uskul et al., 2016) and reduces the tendency to take others’ perspectives (see Galinsky et al., 2016, for a review).

In interpersonal relations, those in power are often less invested (Righetti et al., 2015) and are spontaneously inclined to stand their ground when challenged (Weick et al., 2017). For example, fleeting experiences of high (vs. low) power can cause individuals to confront interaction partners who seek to impose themselves through their non-verbal behavior (Weick et al., 2017). Similarly, testosterone, a substance found in greater concentration in powerful individuals (Dabbs and Dabbs, 2000), exacerbates individuals’ striving for interpersonal dominance (Mazur and Booth, 1998; Pfattheicher, 2016; but see Eisenegger et al., 2010, for conflicting evidence). Dominance and aggression, in this view, provides a route for powerholders to exert influence and reinforce their hierarchical standing, but also to set an example and signal to others to fall in line (Mooijman et al., 2015).

Taken together, the circumstances and behavioral tendencies engendered by high levels of power should, in theory, provide a breeding ground for conflict and aggression, which we define here as *competitive or opposing actions* (Conflict, n.d.) and as the *tendency to cause physical or psychological harm* (Berkowitz, 1993; Bushman and Anderson, 2001), respectively. For example, conflict and aggression increase to the extent that people do not care about the consequences of their actions, have less regard for others, and fail to take others’ perspectives into account (Bandura, 1973; Richardson et al., 1994, 1998; Galinsky, 2002; De Wied et al., 2007)—all established consequences of power as we reviewed above. This negative view of the consequences of power is also reflected in beliefs and expectations that aggression is often directed downward (Moon et al., 2018), which may be linked to the presumption that powerholders do not face any penalties for their actions (Mondillon et al., 2005). However, as discussed below, this negative view of power is often a poor reflection of reality.

It is important to pause and also reflect on the rationale for discussing conflict alongside aggression in the context of power relations. Four out of five common responses to conflict involve aggression (Straus et al., 1996). At the workplace, meta-analytic evidence indicates that conflict is a strong predictor of aggression (Hershcovis et al., 2007). In children, conflict correlates with frequency of aggression to such an extent that the two constructs are essentially indistinguishable (0.69 < *r*s < 0.85; Shantz, 1986). Thus *conflict* describes a situation where people’s thoughts and actions are pitted against others’ thoughts and action, and this situation more often than not translates into aggression.

### How Power Facilitates Conflict and Aggression *in Practice*

Empirically the influence of power on conflict and aggression is not as clear-cut as one might assume. For example, studies on romantic relationships sometimes find high power, and sometimes low power, to be associated with conflict and aggression (Rogers et al., 2005; Bentley et al., 2007). In other social settings, powerholders only appear to aggress against others when they are predisposed to do harm (Chen et al., 2001), or when their position is insecure or unstable (Georgesen and Harris, 2006; Strelan et al., 2014). This suggests that power may only foster conflict and aggression in some circumstances.

Indeed, power heightens aggression when it coincides with greater levels of self-perceived incompetence (Fast and Chen, 2009). Similarly, individuals occupying influential roles that do not command respect and admiration are more inclined to exhibit demeaning behaviors toward others; such behaviors are absent when individuals occupy roles that command respect and admiration, or non-influential roles (Fast et al., 2012). Subsequent studies replicated and extended these findings, showing that high power and low social worth (i.e., *status*) combine to predict conflict in work settings (Anicich et al., 2016).

Taken together, although power may confer a propensity to aggress and impose oneself on others, there is little evidence for a direct link between power and aggression in the absence of moderating factors. Aggression often stems from concerns about one’s worth (Baumeister et al., 1996), which appears to be a key ingredient for triggering negative responses in powerholders. For example, boosting individuals’ self-esteem counters aggression in powerholders who lack competence (Fast and Chen, 2009). Similarly, increasing powerholders’ perceptions of social worth (i.e., *status*) contributes to reduced interpersonal conflict at the workplace (Anicich et al., 2016). This is consistent with the view that powerholders who enjoy others’ respect and admiration do not need to resort to using force and intimidation to influence others (Cheng et al., 2013). Thus, perceived worth seems to play a critical role in the link between power and aggression/conflict.

### The Forgotten Link: Power Boosts Individuals’ Perceived Worth

**FIGURE 1:**
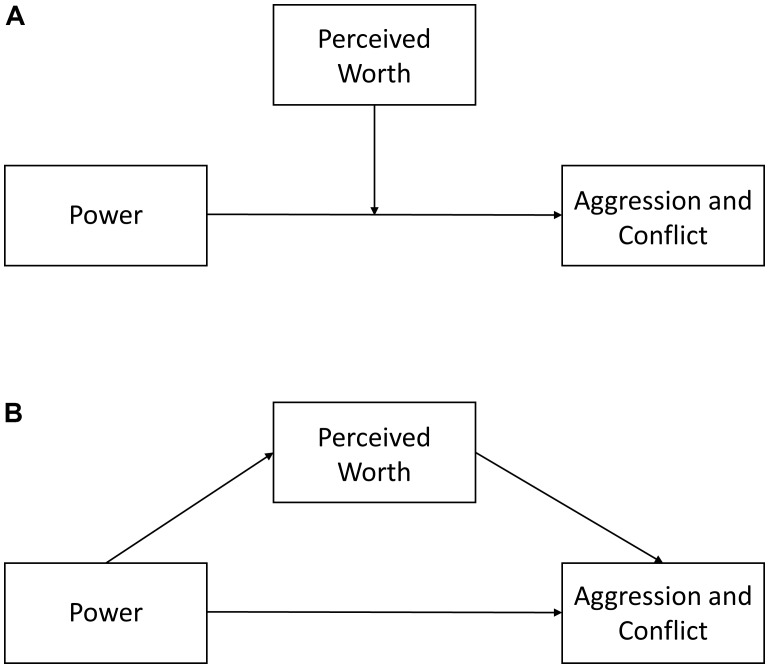
Relation between power, and aggression and conflict, moderated **(A)** and mediated **(B)** by perceived worth.

Previous work has treated powerholders’ perceived worth as a factor that is independent of power, focusing on how power combined with a lack of perceived worth may foster aggression and conflict (**Figure 1A**). This perspective overlooks the fact that being respected by others (i.e., having high status or social worth) is conducive to upward mobility (Fragale et al., 2011; Anderson et al., 2015). What is more, power frequently confers admiration and respect in the eyes of others (Magee and Galinsky, 2008) and bolsters perceptions of competence and efficacy, thereby providing a means to exert influence over others (Sande et al., 1986; Fiske, 2010; Anderson et al., 2012a). Perhaps not surprisingly, then, power boosts self-esteem (Wojciszke and Struzynska-Kujalowicz, 2007), in keeping with theoretical perspectives positing that self-esteem functions as a monitor to track one’s social worth (Leary and Baumeister, 2000; Mahadevan et al., 2016, 2018). Thus, self-esteem and status are closely related constructs in the context of power, and reflect the extent to which powerholders feel liked and respected.

Perceptions of worth reduce the need to assert oneself through coercion (Tesser, 2001; Donnellan et al., 2005; Cheng et al., 2013) and render individuals less sensitive to threats to the self (Harmon-Jones et al., 1997; Green et al., 2008; Sivanathan and Pettit, 2010). If having power provides greater scope for conflict and aggression, yet at the same time boosts individuals’ perceived worth, then the end-result could be a null effect (i.e., the absence of co-variation between power and aggression/conflict), similar to what has been observed in previous work. Stated otherwise, the heightened sense of worth that accompanies power may counter aggression/conflict tendencies in powerholders (**Figure 1B**). Statistically, this *suppression hypothesis* implies that power exerts a (negative) indirect effect on aggression/conflict via perceived worth, which counters the (positive) direct effect of power on aggression/conflict (MacKinnon et al., 2000). The suppression hypothesis is consistent with literature showing that the destructive quality of power arises from a combination of high power and low perceived worth. However, the suppression hypothesis extends previous accounts of power and aggression by taking into consideration the link between power and perceived worth (Fast and Chen, 2009; Fast et al., 2012; Anicich et al., 2016). Stated differently, we maintain that baseline differences in low and high power individuals’ perceived worth are psychologically and behaviorally meaningful and need to be considered toward understanding of the link between power on the one hand, and aggression and conflict on the other.

We hasten to add that not all facets of perceived worth may suppress powerholders’ aggression tendencies—an issue to which we return more fully in the section “General Discussion.” To foreshadow our exposition, studies show that both *low* levels of global self-worth and *high* levels of threatened egotism associated with exaggerated pride are conducive to aggression (Diamantopoulou et al., 2008). In our empirical work reported below, we focus on the benefits of perceived worth for stifling aggression/conflict tendencies, leaving it for future research to address factors that mediate the destructive effects of social power.

We also acknowledge that the link between perceived worth—as measured by self-esteem—and aggression has not been without controversy (Bushman et al., 2009). However, large-scale cross-sectional surveys (Papadakaki et al., 2009; Van Zalk and Van Zalk, 2015), longitudinal surveys (Orth and Robins, 2014), as well as studies of offenders (Garofalo et al., 2016), give considerable credence to the notion that perceived worth stifles, and perceived worthlessness exacerbates, interpersonal frictions.

### The Present Research

The aim of our research was to provide a more complete account of how power contributes to aggression and conflict, taking into consideration the relation between power and perceived worth (**Figure 1B**). To this end, we conducted two studies focusing on *self-esteem* (Study 1) and *status* (Study 2) as two interlinked facets of perceived worth (Leary et al., 1995; Mahadevan et al., 2016, 2018), and examining the contributions of these variables in buffering the influence of power on aggression and conflict—a *suppression* hypothesis.

In Study 2, we report primary data that provide a replication and extension of Anicich et al.’s (2016) Studies 1 and 4, which measured power, status, and conflict. Anicich et al.’s (2016) analyses focused on the interactive effects of power and status depicted in **Figure 1A**. In contrast, in Study 2 reported below, we re-examine and extend Anicich et al.’s results and combine them with our own primary data in a meta-analysis to test our novel suppression hypothesis (**Figure 1B**). We adopt a meta-analytic approach to provide a further test of our model and to enhance the robustness of our findings, in keeping with a cumulative perspective on scientific discovery (Cumming, 2014).

We determined a priori sample sizes for all primary studies. Further, studies were adequately powered (1-β > 80%) to test our theoretical model and probe medium (Study 1) and small-to-medium sized (Study 2) associations between the study variables. However, sample sizes were insufficient to provide precise estimates of population parameters—a task reserved for future research (Schönbrodt and Perugini, 2013).

We carried out data collection in accordance with recommendations of the British Psychology Society Code of Ethics and Conduct. All participants provided written informed consent as per the Declaration of Helsinki. The studies and protocols were approved by the Ethics Committee of the School of Psychology, University of Kent (IDs: 20101399 and 20111576).

## Study 1

In Study 1, a correlational investigation, we focused on self-esteem as an indicator of perceived worth. Following examples from previous work (Donnellan et al., 2005, Study 3; Fast and Chen, 2009, Study 1), participants completed standard measures of power, self-esteem, and aggression. We hypothesized that power would be associated with heightened self-esteem, which in turn would counter aggression tendencies. Put differently, we hypothesized that controlling for the negative indirect effect of power via self-esteem would unveil a positive (direct) relation between power and aggression, which would otherwise be masked by self-esteem acting as a suppressor (MacKinnon et al., 2000).

### Method

#### Participants and Design

One hundred adult volunteers (70 females; *M*_age_ = 21.73, *SD*_age_ = 2.82) from the University of Kent took part in this study. We recruited them through opportunity sampling in the library and other public areas on campus. We offered no payment or course credit. Most participants were students (96%) and enrolled in non-psychology degree programs (93%); 75% were Caucasian, 11% Black, 9% Asian, and 5% had a mixed ethnic background.

#### Procedure and Materials

An experimenter unaware of the hypothesis instructed participants to complete a questionnaire booklet that contained all study materials, the order of which we counterbalanced. We assessed power with an 8-item scale (‘*I can get others to do what I want’*; 1 = *disagree strongly* to 7 = *agree strongly*; Anderson et al., 2012b). We assessed perceived worth with Rosenberg’s (1965) 10-item self-esteem scale (‘*I take a positive attitude toward myself*’; 1 = *disagree strongly* to 7 = *agree strongly*). Lastly, we assessed aggression with the 12 item Short-Form Buss-Perry Aggression Questionnaire (‘*Given enough provocation, I may hit another person’*; 1 = *not at all characteristic of me* to 6 = *extremely characteristic of me;* Bryant and Smith, 2001).

### Results and Discussion

**FIGURE 2:**
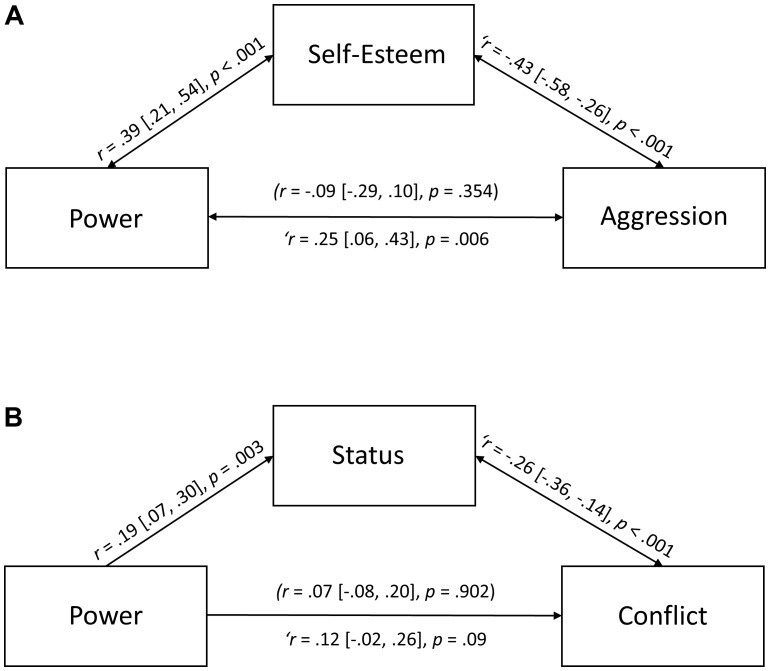
Perceived worth (**A**: self-esteem; **B**: status) buffering the influence of power on aggression and conflict.

We created single indices of power (α = 0.88, *M* = 4.83, *SD* = 0.82), self-esteem (α = 0.79, *M* = 5.15, *SD* = 1.11), and aggression (α = 0.84, *M* = 2.67, *SD* = 1.04).^1^ As anticipated, power shared no overall (i.e., zero-order) relation with aggression, *B* = 0.12 [-0.13, 0.37], *SE* = 0.13, *t*(98) = 0.93, *p* = 0.354, *r* = -0.09 [-0.29, 0.10]. (We provide 95% confidence intervals in square brackets for regression coefficients and effect sizes, respectively). Meanwhile, power was positively related to self-esteem, *B* = 0.29 [0.15, 0.42], *SE* = 0.07, *t*(98) = 4.16, *p* < 0.001, *r* = 0.39 [0.21, 0.54], and self-esteem was negatively related to aggression, *B* = -0.34 [-0.52, -0.17], *SE* = 0.09, *t*(98) = -3.86, *p* < 0.001, *r* = -0.36 [-0.52, -0.18]. The indirect effect of power on aggression via self-esteem was significant, *Z*_Sobel_ = -3.17, *p* = 0.002.^2^ To find out if the (negative) indirect effect masked a positive (direct) effect of power on aggression, we regressed aggression scores on the measures of power and self-esteem; both power (*B* = 0.35 [0.10, 0.60], *SE* = 0.13, *t*(97) = 2.79, *p* = 0.006, *r*_semi-partial_ = 0.25 [0.06, 0.43]) and self-esteem (*B* = -0.44 [-0.63, -0.26], *SE* = 0.09, *t*(97) = -4.76, *p* < 0.001, *r*_semi-partial_ = -0.43 [-0.58, -0.26]) emerged as significant predictors. Given that the (negative) indirect effect is directionally opposite to the (positive) direct effect of power on aggression controlling for self-esteem, the overall pattern of results in consistent with a suppression effect. In other words, elevated self-esteem countered the positive association between power and aggression (**Figure 2A**).

These findings provide, for the first time, an indication that power-associated boosts in self-esteem may mitigate the negative influence of power on aggression. At the same time, the correlational nature of the data makes it impossible to ascertain the direction of the observed effects (Fiedler et al., 2018). For example, we are in no position to rule out that aggression (as predictor) had a positive direct effect on perceptions of power (as outcome), and a negative indirect effect via self-esteem (as mediator/suppressor), which would be akin to flipping **Figure 1B** on a horizontal axis. Establishing the causal direction of our suppression model necessitates an experimental approach (Fiedler et al., 2018), manipulating different levels of power and assessing variations in the mediator/suppressor and in the outcome variables. Hence, in Study 2, we adopted the said experimental approach. It is worth noting that the present findings are situated in an extant literature, which shows that manipulating different levels of power leads to variations in the mediator/suppressor (self-esteem), but it does not affect the outcome variable (aggression) in absence of other moderating factors (Wojciszke and Struzynska-Kujalowicz, 2007; Fast and Chen, 2009).

Our previous study used self-esteem as a marker of worth, which is linked to one’s social standing (Leary and Baumeister, 2000; Mahadevan et al., 2016, 2018). In our next study, we examine status as a marker of social worth in a group setting. We also focus on *conflict* as a broader construct that allows examination of how perceived worth buffers against the deleterious interpersonal consequences of power.

## Study 2

In Study 2, an experimental investigation, participants took part in small group interactions, and were assigned to a high power or low power role. We hypothesized that, relative to those in a low power role, participants in a high power role would experience greater status in the group, which in turn would buffer against conflict. Put otherwise, we expected to observe a negative indirect effect of power on conflict via status, which would mask a positive direct effect of power on conflict.

As noted earlier, the present study provides a replication and extension of Anicich et al.’s (2016) Studies 1 and 4, which measured power, status, and conflict.^3^ Here, we re-examine Anicich et al.’s results to test our suppression hypothesis (**Figure 1B**). Below, we first report the outcome of our primary research before moving on to a meta-analytic synthesis of all primary and secondary data (for a similar approach, see: Leach et al., 2017; Leach and Weick, 2018). For ease of reference, we also provide brief summaries of the methods employed by Anicich et al. (2016).

### Method

#### Participants and Design (Primary Data)

Two hundred and sixty adults (220 females; *M*_age_ = 19.55, *SD*_age_ = 2.82^4^) recruited from the University of Kent took part in return for course credits or a monetary incentive (∼$25). All participants were students, with 67% majoring in psychology. Also, 83% identified their ethnicity as Caucasian, 6% as Asian, 6% as Black, and 5% as mixed.

Participants were assigned to one of 65 same-sex, same-ethnicity, 4-member groups. Within each group, two participants were randomly assigned to a high power role (managers) and two to a low power role (assistants).

#### Participants and Design (Secondary Data)

Anicich et al. (2016) gathered data from 86 adults recruited from a national database in Study 1 (53 women; *M*_age_ = 37.84, *SD*_age_ = 10.53), and 128 employees from a federal agency in Study 4 (38 females; *M*_age_ = 45.53, *SD*_age_ = 9.13).

#### Procedure and Materials (Primary Data)

We conducted the study in a controlled, laboratory environment. Upon arrival, an experimenter unaware of the hypothesis instructed participants that they would perform tasks in groups of four. Following a short introduction to the other group members, two participants were randomly assigned to the role of managers, and two to the role of assistants, by means of a lottery. The managers’ task was to pass on instructions to the assistants and to oversee the assistants’ work. To ascertain the success of the power manipulation, participants indicated *how much in charge* they were in the group task, and *how much influence* they had over the other team members (1 = *not at all*, 9 = *very much*). All participants then met for approximately 40 min to work on a series of unrelated creative problem exercises, similar to those commonly used in creativity research (Wallach and Kogan, 1965).^5^ The two managers took turns instructing the two assistants and monitoring the time allotted to each group task. After the exercises, participants returned to individual rooms, where they completed a questionnaire evaluating their interactions. Two 7-point scales assessed participants’ status in the group: ‘*To what extent did the other group members ‘look up’ to you?’*, and ‘*What was your status in the group?’* (1 = *not at all*, *very low* to 7 = *very much, very high*). In addition, four items adapted from Janssen et al. (1999) assessed perceived conflict with other group members who occupied a different role: ‘*The personal relationship with the managers [assistants] was excellent*,’ ‘*I did not get on personally with the managers [assistants]*,’ *‘I regularly took divergent viewpoints on the issues involved,’ ‘I had often very different ideas than the managers [assistants] in important matters’* (1 = *extremely inaccurate* to 7 = *extremely accurate*). In an exploratory practice, we asked participants to respond to these items both from their own perspective (‘*I did not get on with the managers [assistants]* and from the perspective of the other group members (‘*The managers [assistants] did not get on with me*’).

#### Procedure and Materials (Secondary Data)

Anicich et al. (2016) asked participants to indicate whether they had the authority to *hire/fire others*, which served as a dichotomous measure of power in Study 1, or whether employees had *control over valuable resources that others in the organization need and/or the ability to administer rewards and punishments*, which served as a single-item measure of power in Study 4. To measure status, they asked participants to respond to four items in Study 1 (e.g., ‘*To what extent does your position at work give you high status in the eyes of others*?; α = 0.68), and a single-item scale in Study 4 (the amount of ‘*respect, admiration, and prominence you possess in the eyes of others*’). Finally, they assessed conflict with three items such as ‘*I often have personal disagreements with others at my place of work*’ (α = 0.92) in Study 1, and with four items such as ‘*How frequently are there conflicts about ideas among people you work with*?’ (α = 0.87) in Study 4.

### Results (Primary Data)

For the manipulation check, the response of one participant was missing, and so we substituted it with the mean response of the other team-members in the same role (manager). As individuals’ responses (level 1) were nested within teams (level 2), we used multi-level modeling to estimate random intercepts and slopes wherever possible and irrespective of whether random effects were significant or not, in order to counter Type I error inflation (Barr et al., 2013). Only where model estimates did not converge, we excluded random effects. To facilitate results presentation, our discussion focuses on fixed effects obtained using REML. We derived effect sizes from *t*-values and obtained degrees of freedom via Satterthwaite’s approximation. As in Study 1, we provide 95% confidence intervals in square brackets.

#### Manipulation Check

We regressed perceived power (*r* = 0.89, *M* = 5.67, *SD* = 2.48) on a dummy variable representing the two power conditions (low power: *D*_1_ = 0, high power: *D*_1_ = 1), which confirmed that the managers (*M* = 7.78, *SD* = 0.89) felt they had more power than the assistants (*M* = 3.57, *SD* = 1.61), *coeff* = 4.21 [3.90, 4.53], *SE* = 0.16, *t*(258) = 26.14, *p* < 0.001, *r* = 0.85 [0.82, 0.88]. The manipulation was effective.

#### Main Analysis

At first, we created single indices of status (α = 0.79, *M* = 5.15, *SD* = 1.11) and conflict (α = 0.78, *M* = 2.74, *SD* = 0.84) after reverse coding negatively worded items. We then proceeded to examine the overall (i.e., zero-order) relation between power and conflict. In keeping with Study 1, there was no indication that team members in high power roles (*D*_1_ = 1) got on less well with others compared to team members in low power roles (*D*_1_ = 0), *coeff* = 0.09 [-0.11, 0.29], *SE* = 0.10, *t*(194.00) = 0.90, *p* = 0.368, *r* = 0.07 [-0.08, 0.20]. However, participants in a high power role felt they had more status than participants in a low power role, *coeff* = 0.37 [0.08, 1.14], *SE* = 0.12, *t*(258.00) = 3.04, *p* = 0.003, *r* = 0.19 [0.07, 0.30], and the higher the status of team members in the group, the less they experienced conflict in their team, *coeff* = -0.20 [-0.30, -0.10], *SE* = 0.05, *t*(250.18) = -3.92, *p* < 0.001, *r* = -0.24 [-0.35, -0.12]. A Sobel test confirmed that the negative indirect effect of power on conflict via perceived status was significant, *Z*_Sobel_ = 2.46, *p* = 0.014. In a final step, we sought to isolate the direct effect of power on conflict, controlling for the indirect effect of power via status. To this end, we regressed conflict scores on both power and status. The results revealed that elevated status stifled conflict (*coeff* = -0.22 [-0.32, -0.11], *SE* = 0.05, *t*(250.00) = -4.19, *p* < 0.001, *r*_semi-partial_ = -0.26 [-0.36, -0.14]), whereas elevated power exacerbated conflict (*coeff* = 0.17 [-0.03, 0.37], *SE* = 0.10, *t*(195.78) = 1.72, *p* = 0.088, *r*_semi-partial_ = 0.12 [-0.02, 0.26]), although the latter effect was marginal. All in all, the results are consistent with our suppression model (**Figure 2B**).

As a means of providing a further critical test of our results, we also examined an (unpredicted) conceptual model whereby power (*X*) impacts perceived status (*Y*) via conflict (*Z*). This yielded no evidence for suppression or mediation; *Z*_Sobel_ = 0.88, *p* = 0.378, for the indirect pathway. Note that, because power was manipulated experimentally, there are no other viable alternatives to describe the causal relation among power, status, and conflict.

### Results (Meta-Analysis)

In a final step, we sought to establish the robustness of our findings through a meta-analysis that incorporates a re-examination of Anicich et al.’s (2016) results (Cumming, 2014). First, we calculated effect sizes (*r*) for the overall (i.e., zero-order) association between power and conflict, for the association between power and perceived worth (status), and for the unique associations (semi-partial *r*) between power and perceived worth on the one hand, and conflict on the other. We compiled this information from statistics reported in Anicich et al. (2016). We then conducted a fixed effects meta-analysis to derive estimates of the combined effect sizes (*n*_total_ = 474). As can be seen in **Table 1**, power shared no zero-order relation with conflict, *r*_combined_ = 0.01, *p*_combined_ = 0.772. However, elevated power was associated with increased status, which in turn shared a negative association with conflict. The indirect (negative) effect of power on conflict via status was significant at the meta-level, *Z*_Sobel_ = 4.01, *p* < 0.001. Furthermore, controlling for variations in status unveiled a (positive) direct association between power and conflict, *r*_combined_ = 0.10, *p*_combined_ = 0.067. Thus, the suppression pattern observed in our primary research appears to be indicative of a more general phenomenon.

**Table 1:** Study-level and meta-level pathways predicting conflict from power and status (Study 2).

		Power -> status		*‘*Status -> conflict		Power -> conflict		*‘*Power -> conflict	
Data source	*n*	*r*	*p*	*‘r*	*p*	*r*	*p*	*‘r*	*p*
	Study level								
Primary	260	0.19	0.003	-0.26	<0.001	0.07	0.368	0.12	0.088
Secondary (Anicich et al., 2016, Study 1)	86	0.37	<0.001	-0.43	<0.001	0.10	0.363	0.27	0.013
Secondary (Anicich et al., 2016, Study 4)	128	0.62	<0.001	-0.09	0.319	-0.16	0.076	-0.07	0.415
	Meta level								
	474	0.36	<0.001	-0.25	<0.001	0.01	0.772	0.10	0.067

*‘r = semi-partial correlation coefficient. All p-values are two-tailed. For the fixed effects meta-analysis, studies are weighted by sample size (n).*

## General Discussion

Those who wield power are thought to be more likely to aggress and spur conflict than those who do not wield power (Mondillon et al., 2005; Moon et al., 2018). However, contrary to this pervasive supposition, such a relation between power and aggression/conflict has not been borne out in the empirical literature (Rogers et al., 2005; Georgesen and Harris, 2006; Bentley et al., 2007; Fast and Chen, 2009; Fast et al., 2012; Strelan et al., 2014). We hypothesized that one reason for this null effect is the buffering influence of heightened worth that often accompanies power, which provides a buffer against threats to the self (Green et al., 2008) and reduces the need to assert oneself through coercion (Cheng et al., 2013). The results of two studies were consistent with this hypothesis. In Study 1, we found a positive (direct) association between power and aggression. However, this association was suppressed and indeed nullified by perceived worth (here: *self-esteem*), which shared a positive association with power, and a negative association with aggression. In Study 2, we conceptually replicated this finding. Drawing on primary and secondary data, we obtained evidence for perceived worth (here: *status*) acting as a suppressor and countering an otherwise positive association between power and conflict.

Prior work has indicated that aggression and conflict are likely to ensue when powerholders’ worth is threatened (Georgesen and Harris, 2006; Fast and Chen, 2009). The present findings fit with and extend prior work by demonstrating that the direct positive relation between power and aggression/conflict fails to emerge due to the suppressing effects of perceived worth. In theoretical terms, the findings highlight that the association between power and perceived worth is critical for a full understanding of the link between power on the one hand, and aggression and conflict on the other.

In methodological terms, our findings highlight the importance of considering suppression mechanisms in the phenomena with which social and personality psychologists are concerned. Although studies involving moderator and mediator variables seeking to explain the influence of one variable on another abound, suppression processes are often overlooked. Yet, as our research revealed, the absence of an association between two or more variables can mask meaningful psychological processes with possible implications for real-life settings.

### Limitations

The association between power and conflict controlling for status was small overall (*r* = 0.10). As shown in **Table 1**, this is primarily due to Study 4 of Anicich et al. (2016), where the association between status and conflict was not significant. This pattern of results may be due to the way conflict was measured, referring more to people’s workplace in general rather than their own relationships with others (e.g., ‘*One party frequently undermines another*,’ ‘*There are often feelings of hostility among parties*’). There is a need for further research into the suppression effect identified here, extending the scope of the meta-analysis beyond the three data-sets that we were able to synthesize. Future empirical efforts should also incorporate behavioral measures, going beyond the self-reports employed in the present work. On a related note, as only one of our studies provided experimental evidence, further research is needed to probe the causal pathways depicted in our conceptual model (**Figure 1B**).

Finally, our approach, representing a foray into these issues, was somewhat haphazard, as Study 1 measured cross-sectionally power, self-esteem, and aggression (but not conflict), whereas Study 2 (primary data) manipulated power and assessed status and conflict (but not aggression). Future investigations would benefit from adopting a more systematic approach in the manipulation and measurement of the corresponding constructs.

### Implications

A good deal of research has documented the benefits of perceived worth for the individual (see Anderson et al., 2015, for a review). The current work adds to a small body of evidence highlighting the benefits of perceived worth in interpersonal and inter-group relations. For example, Gregg et al. (2018) found that, although in general people derogate (i.e., evaluate as less intelligent and moral) their ideological opponents, higher status—measured or manipulated—moderated this effect. That is, higher status was associated with, or led people to, a reduction in opponent derogation. The Gregg et al. (2018) results dovetail with the current findings, indicating that higher status, reflecting social worth, can curtail the tendency to behave aggressively toward potentially threatening others (see also Henry, 2009).

The present findings align with recent evidence that power and status can have distinct, and in some cases opposing, consequences for individuals (Blader and Chen, 2012; Blader et al., 2016). However, the present investigation also highlights the drawbacks of conceptualizing power and status as independent constructs. Arguably, such a conceptualization provides an incomplete reflection of hierarchical relationships in real life where status and power tend to co-vary (Fiske et al., 2016), masking meaningful psychological processes such as the suppression mechanisms identified herein.

Related to the previous point, the current findings speak to an emerging literature that highlights the social regulatory function of powerholders’ feeling liked and respected. For example, collaborations between powerful individuals are hampered by status conflicts, thereby worsening the performance of the group as a whole (Hildreth and Anderson, 2016). This suggests that allocating respect and admiration to (selected) decision-makers within a group can lead to better coordination in groups and smoothen out interpersonal relations.

Through strategies, such as pleasing and buttressing others’ reputation, ingratiators can of course also achieve positive outcomes for themselves (Gordon, 1996; Higgins et al., 2003). However, our work suggests that in hierarchical relationships ingratiation may serve a more fundamental purpose by enabling low power ingratiators to avert negative outcomes. This would be akin to dynamics observed in the primates’ literature, where ‘grooming’ fulfills similar rank-related functions and renders powerful animals more tolerant and less likely to aggress (Henazi and Barrett, 1999; Schino, 2001). Interestingly, low power (human) ingratiators may need to tread a fine line, because they risk a backlash if their attempts to please powerholders are too blunt (Liden and Mitchell, 1988; Inesi et al., 2012; Steinmetz et al., 2017).

Boosts in perceived worth (self-esteem or status) may also have other negative consequences. Let us consider the case of narcissism, a dominant, self-aggrandizing, and manipulative social orientation. Narcissists are high in need for power, are antagonistic, and respond aggressively to those who criticize or outperform them (for reviews, see: Sedikides and Campbell, 2017; Thomaes et al., 2018). A rise in perceived worth (e.g., self-esteem) might exacerbate, rather than soothe, these ills. Thus, elevations in perceived worth may backfire in the case of narcissists. Future research would do well to test the boundaries (personality and beyond) of the effects we identified in the current work.

### Coda

In ‘King Lear,’ William Shakespeare noted that powerful individuals bow to flattery. Altering powerholders’ perceived worth could indeed be a way for subordinates to avert harm and exert upward influence in hierarchies. It remains for future empirical endeavors to uncover the full scope of the regulatory function of perceived worth (self-esteem and status) in social hierarchies.

## Author’s Note

The conceptual model and narrative interpretations of empirical data appearing in this article were presented by MW as part of an internal academic seminar series at the School of Psychology, University of Kent, on June 4th, 2013.

## Author Contributions

MW designed the studies, oversaw the data collection, and performed the data analysis. All authors contributed to the conceptual work, writing of the manuscript, and approved the final version of the manuscript.

## Conflict of Interest Statement

The authors declare that the research was conducted in the absence of any commercial or financial relationships that could be construed as a potential conflict of interest.

## Footnotes

1 We ascertained that the results cannot be attributed to artifacts or outliers by examining the distribution of raw scores and the normal probability plot of residuals. We also replicated the observed pattern of results using robust regression analyses.

2 Using an alternative bootstrapping technique to establish the significance of the indirect effect yields the same results (Hayes, 2017). For consistency, we adopt the Sobel test, as Study 2 involves statistical techniques (i.e., multi-level modeling, meta-analysis) for which bootstrapping options to probe indirect effects are not readily available. Note that the Sobel test is usually more conservative than Preacher and Hayes’s (2004) bootstrapping approach (Zhao et al., 2010).

3 Studies 2 and 3 reported in Anicich et al. (2016) manipulated power and status orthogonally, thereby nullifying the (positive) association between power and status that one would normally expect to observe and that in our theoretical model gives rise to a suppression effect.

4 Age information from one participant was missing and imputed with the sample mean.

5 Materials for the creativity task are available from the authors on request.
